# Exploring the Role of Social Factors in Cognitive Frailty among South Korean Older Adults

**DOI:** 10.3390/healthcare12141394

**Published:** 2024-07-11

**Authors:** Young Ko, Kyungwon Choi

**Affiliations:** 1 College of Nursing, Gachon University, Incheon 21936, Gyeonggi, Republic of Korea; youngko@gachon.ac.kr; 2 Department of Nursing, Korea National University of Transportation, Jeungpyeong-gun 27909, Chungbuk, Republic of Korea

**Keywords:** cognition, frailty, social factor, older adults, community

## Abstract

Cognitive frailty, which is characterized by the co-occurrence of physical frailty and cognitive impairment, poses significant risks to the well-being and independence of elderly individuals. Previous research has established that demographic, health-related, and social factors contribute to both physical frailty and cognitive decline. However, the role of social factors in influencing cognitive frailty remains unclear. This study aims to identify the relationship between social factors and cognitive frailty among Korean older adults living in the community. We performed secondary analyses of data from the 2020 Survey on Older Adults. After entering demographic factors and health-related factors into the logistic model as covariates, this study explored the association between cognitive frailty and social factors, including living arrangements, social support, the frequency of engagement in social activities per week, and satisfaction with friends and community. Among participants, approximately 2.9% had cognitive frailty, 3.2% had only physical frailty, and 21.9% had only cognitive decline. Lower levels of satisfaction with friends and the community and infrequent participation in social activities were strongly correlated with cognitive frailty. These findings emphasize the necessity of public health programs that encourage older individuals’ social involvement. A supportive social environment can be fostered through initiatives that promote community events, group activities, and volunteerism. Public health policies should prioritize the development and maintenance of social activity centers that offer various programs to prevent progression to cognitive frailty in older adults.

## 1. Introduction

South Korea is currently experiencing a rapid transition into an aging society. These changes are being caused by a significant decline in birth rates and increases in average life expectancy due to healthcare advances, economic pressures such as high costs of living and child-rearing, evolving social norms, and individualistic lifestyles [1]. The prevalence of aging-related diseases and frailty has continued to grow with an increase in the elderly population. Frailty, characterized by a decline in the physiological reserve capacity of various organs among older adults, manifests as diminished resilience to stressors, thereby rendering individuals more susceptible to negative health outcomes such as disability, illness, hospitalization, and mortality [2,3]. Western countries, particularly in Europe and North America, tend to have more individualistic cultures. Elderly care is often institutionalized, with greater reliance on nursing homes and assisted living facilities. In contrast, Korea’s collectivist culture emphasizes family-based care. The increase in women’s labor force participation and nuclear families is making it more difficult for families to care for the elderly [4]. These changes require greater public health attention to the prevention and management of frailty among the elderly as well as the establishment of a community support system for elderly care.

Frailty is a reversible state before reaching irreversible adverse health outcomes such as disease or death [5]. Therefore, frailty is an important concept in that appropriate interventions can maintain and improve function to prevent disease or death. While frailty encompasses multidimensional aspects, including physical, cognitive, emotional, and social functions [6], previous research has predominantly concentrated on physical frailty. In general, the most commonly used definition of frailty is Fried’s phenotype, which delineates the condition through the presence of three or more of the following five criteria: unintentional weight loss, exhaustion, weakness, slow gait speed, and low levels of physical activity [2]. A meta-analysis study carried out to assess the prevalence of frailty in the Chinese community has reported that the prevalence of physical frailty varied from 5.9% to 17.4%, with a pooled prevalence estimated at 10% [7]. In a multicenter longitudinal study conducted in 2016–2017, physically frail Korean older adults accounted for 7.8% of community-dwelling residents in urban and rural areas nationwide in 10 study centers across different regions, and this figure is expected to steadily increase [8]. Therefore, it is important to periodically monitor the incidence of frailty, including physical frailty, and reduce frailty-related risk factors to mitigate the prevalence of frailty among the older population. Cognitive impairment also increases with age. The prevalence of cognitive impairment has varied from about 5.1% to 41%, with a median of 19.0% [9]. These cognitive impairments elevate the susceptibility to physical and functional impairment, dementia, frailty, and death [10,11,12,13]. Previous studies have found that cognitive impairment and physical fragility are associated [14] and that there is an increased risk of physical frailty in the presence of cognitively impaired people [11,12,15]. This implies a complex interplay between cognitive and physical frailty domains within the aging-related degenerative trajectory [16]. There is a strong link between being physically weak and losing your mental abilities. This link is based on similar mechanisms, such as changes in vascular and hormonal function, a lack of certain nutrients and vitamins (especially vitamin D and B12), inflammation, and insulin resistance [17].

Cognitive frailty, an emerging concept in the field of geriatric medicine, has garnered increasing attention from scholars and healthcare professionals in recent years [18]. This condition is characterized by the coexistence of physical frailty and cognitive decline [18]. Individuals affected by cognitive frailty experience a spectrum of health challenges, ranging from cognitive impairment to functional limitations, ultimately leading to a decreased quality of life and elevated mortality rates compared to their healthier counterparts [19,20]. Given these implications, it is important to identify both the prevalence and characteristics of frailty, with a specific focus on cognitive frailty.

Demographic factors that have been associated with physical frailty [21] and cognitive impairment [22] include female gender, advanced age, low economic status, low educational attainment, and single or unpartnered status. Health characteristics that have been associated with physical frailty [21] and cognitive impairment [22] include dependency on daily activities, decline in vision and hearing function, the presence of chronic diseases, depressive symptoms, and poor self-rated health. Furthermore, studies have found that social factors influence the physical frailty [21,23,24,25] and cognitive function [26] of older adults. However, previous studies examining the relationship between social factors and frailty produced inconsistent findings. Instrumental support from within and outside family members was associated with a significantly lower risk of physical frailty [25]. Similarly, social isolation—measured through marital status, frequency of contact with offspring, and engagement in various social activities—was also associated with the progression of physical frailty [23]. High levels of social activities significantly influenced frailty positively, regardless of cognitive function level [24]. However, some studies have found that too much social support may negatively affect the mental health of older adults [27,28]. Older people may complain of more depression as they receive more types of social support and have less personal control [27]. Similarly, a scoping review highlighted that older adults need a balanced approach to care. Excessive help with activities of daily living, such as bathing and dressing, can lead to decreased independence and lower self-esteem, ultimately worsening their condition [28]. As a result of a systematic literature review, the relationship between functional social support and higher cognitive function in middle- and older-aged adults was not statistically significant [29].

While most studies have not investigated the association between social factors and cognitive frailty, some studies have found that social factors influence cognitive frailty [30,31]. Social support was negatively associated with cognitive frailty [30]. Similarly, traveling or outing experiences less than once a year were associated with cognitive frailty [31].

Based on these findings, we aimed to identify the prevalence of cognitive frailty in older adults residing in the community and identify the factors influencing cognitive frailty, focusing on social factors. We hypothesized that social factors would influence cognitive frailty and that the influence of social factors on the health of older adults would vary depending on the characteristics of social factors.

## 2. Materials and Methods

### 2.1. Study Design and Participants

This study was a secondary analysis that used data from the 2020 National Survey of Older Koreans (NSOK) to identify factors associated with the cognitive frailty of older adults living in communities. The Department of Health and Social Research and the Ministry of Health Welfare have jointly conducted the NOSK every three years since 2008, using a nationally representative sample of older persons aged 65 and above. The Institutional Review Board of the Korea Institute for Health and Social Affairs (KIHSA IRB Number: 2020-36) officially approved this survey [32]. In 2020, they surveyed 10,299 elderly people living in 17 districts and 934 survey areas. The study used data from 9827 older adults without a dementia diagnosis and without any missing information to assess cognitive frailty.

### 2.2. Measure

#### 2.2.1. Cognitive Frailty

Physical frailty and cognitive impairment coexist alongside cognitive frailty [18]. We measured physical frailty using the FRAIL instrument [33], which defines frailty as the absence of problems in fewer than three domains: fatigue, resistance, ambulation, illness, and weight loss [34]. A FRAIL score of 3 or more denotes physical frailty, while scores of 1–2 indicate prefrailty, and a score of 0 signifies robust. We evaluated cognitive function using the Korean version of the Mini-Mental State Examination for Dementia Screening (MMSE-DS) [35]. The score range of MMSE-DS is from 0 to 30. Normal and cognitive impairment are classified according to the norm score based on age, sex, and education level. We identified cognitive impairment as scores that fell 1.5 standard deviations below those expected for the age and education levels of older Korean adults [35]. In this study, mean MMSE-DS was 3 points and ranged from 1 to 5 points. This standardized test has demonstrated reliability and validity for screening cognitive impairment, including dementia. We classified the participants into the following four groups based on the combination of physical weakness and cognitive decline: robust, cognitive impairment only, physical frailty only, and cognitive frailty.

#### 2.2.2. Social Factors

Social factors comprised both structural and functional variables. Structural variables included living arrangements, social support measured by the number of close friends, and relatives, and the frequency of engagement in social activities per week. Functional variables included satisfaction with friends and the community. We categorized living arrangements into two groups: living alone and living with others.

The frequency of social activity participation per week was calculated by summing the frequency of seven social activities per week: club, social club, political and social group, volunteer activity, religious activity, senior citizen’s center, and welfare center for seniors. The frequency of participation in social activities per week was classified into four categories: less than once per week, once per week, 2–3 times per week, and four or more times per week. The assessment of social support involved asking the following question: “How many close relatives, friends, neighbors, and acquaintances, including brothers and sisters, do you have with whom you can confide in your mind?” Satisfaction with friends and the community was assessed using “To what extent are you satisfied with relationships with friends and the community?” Response options included (1) very satisfied; (2) satisfied; (3) average; (4) not satisfied; and (5) not satisfied at all. We grouped responses of “very satisfied” and “satisfied” as “satisfied”, while we grouped “average”, “dissatisfied”, and “very dissatisfied” as “dissatisfied”.

#### 2.2.3. Covariates

Demographic factors, including sex, age, educational attainment, and equivalent household annual income, were considered covariates in this study. We categorized age groups as 65–74 years, 75–84 years, and 85 years older. House income was measured by total income. We then divided the total annual household income by the square root of the total number of household members and classified it according to all quartile distributions (the lowest 25%, 25–50%, 51–75%, and the highest 25%).

Health-related factors included vision, hearing, depression, a number of chronic diseases, and Instrumental Activities of Daily Living (IADL) dependency. Visual and hearing sensory functions were evaluated through inquiries of discomfort experienced in daily life regardless of the use of aids such as glasses and hearing aids. The questions were about discomfort while watching television, reading the newspaper, and talking on the phone or with someone next to them. Responses indicating “not uncomfortable” were classified as “good”, whereas those indicating varying degrees of discomfort, namely “uncomfortable” or “very uncomfortable”, were categorized as “not good”.

IADL was measured using the Korean version of IADL adapted by Won et al. [36], based on the tool developed by Lawton et al. [37]. The scale consists of 10 items (scored as 1 point for complete independence, 2 points for partial help, and 3 points for complete help), with higher scores indicating greater dependence. We classified participants as ‘IADL-dependent’ if they indicated any need for help on any of the 10 items, and as ‘IADL-independent’ if they were completely independent.

Depressive symptoms were assessed using a 15-item scale adapted from the Geriatric Depression Scale (GDS) developed by Sheikh and Yesavage [38], which was translated into Korean and validated by Kee [39]. Each item was rated as “yes” or “no,” with total scores ranging from 0 to 15. Participants with scores of 10 or higher were considered to have depressive symptoms. The scale demonstrated good reliability (Cronbach’s alpha = 0.84).

We assessed the self-reported number of diagnosed chronic diseases (including hypertension, stroke, hyperlipidemia, angina, myocardial infarction, diabetes, thyroid disease, arthritis, neuropeptide, osteoporosis, back pain, chronic bronchitis, emphysema, asthma, tuberculosis, cataracts, glaucoma, chronic otitis media, cancer, gastroduodenal ulceration, hepatitis, liver cirrhosis, chronic kidney disease, benign prostatic hyperplasia, urinary incontinence, sexually transmitted diseases, anemia, skin disease, depression, fracture, insomnia, Parkinson’s disease, etc.).

### 2.3. Data Collection

We used publicly available data that did not contain identification information. Data were obtained from the 2020 National Survey of Older Koreans via the website (https://data.kihasa.re.kr/kihasa/main.html (accessed on 27 July 2021)) after our application was reviewed and approved. The original survey was conducted between 14 September and 20 November 2020. A trained investigator collected data using a tablet-PC-assisted personal interview method. The investigator visited the participants’ homes and directly collected data through interviews [32]. Approval for the study was obtained from the Institutional Review Board of the researchers’ affiliated university (IRB No. 1044396-202404-HR-065-01).

### 2.4. Statistical Analysis

SPSS 26.0 was used for all statistical analyses. The characteristics of demographic factors, health-related factors, family and social factors, and cognitive frailty as well as the correlation between these influencing factors and cognitive function were examined using χ^2^ tests, *t*-tests, a one-way ANOVA with Scheffe tests, and descriptive statistics. Three multiple logistic regression analyses were performed to identify the factors influencing cognitive impairment, physical frailty, and cognitive frailty. For these analyses, the model was run with four demographic factors (sex, age, educational level, and annual household income), three health-related characteristics (IADL dependency, depression, and number of chronic diseases), and family and social factors in association with the status of cognitive frailty. Odds ratios (ORs) indicated the likelihood of membership in the “cognitive impairment” group (relative to the “robust” group), the “physical frailty” group (relative to the “robust” group), and the “cognitive frailty” group (relative to the “robust” group).

## 3. Results

### 3.1. Cognitive Frailty

Among the older adult participants, the prevalence rates were as follows: cognitive frailty at 2.9%, physical frailty at 3.2%, and cognitive decline at 21.9%. About 56.9% of older adults were women, and 5.9% were aged 85 or older. Furthermore, 41.9% had achieved an elementary school or lower level of education. Moreover, 34.7% reported experiencing vision discomfort, and 24.6% reported experiencing hearing discomfort. Participants had an average of 1.83 chronic diseases, 32.1% had depressive symptoms, and 10.8% had IADL dependence. Both demographic and health-related factors were found to be associated with cognitive frailty.

The prevalence of cognitive frailty was higher among women than men and was particularly elevated among individuals with low income and lower levels of education. Moreover, participants with depressive symptoms exhibited a higher prevalence rate of cognitive frailty (7.8%) compared with older adults without depressive symptoms (0.6%). The correlation between physical frailty and cognitive frailty increased with the number of chronic diseases. Additionally, there was a significant correlation between IADL dependence and cognitive frailty (Table 1).

Approximately 20% were living alone, and 80.0% were living with others. About 19.7% engaged in social activities less than once a week, and they reported having an average of 3.05 close acquaintances. Moreover, 59.0% of older adults expressed satisfaction with their friends and community.

Cognitive frailty was associated with all social factors. The number of close people was significantly lower in the physically frail and cognitively frail groups compared to the robust and cognitively impaired groups (*p* < 0.001). Moreover, Table 2 shows a strong correlation between cognitive frailty and lower levels of satisfaction with friends and the community, as well as reduced engagement in social activities.

### 3.2. Factors Influencing Cognitive Frailty

When comparing the cognitive impairment group with the robust group, the likelihood of cognitive impairment increased in those aged 74–84 and over 85 compared to those aged 65–74, those with higher levels of education, or those with a low-income level. Furthermore, participants with hearing discomfort, depressive symptoms, or dependence on IADL were more likely to experience cognitive impairment.

Regarding social factors, the odds of cognitive impairment were 1.40 (1.23–1.61) times higher in older adults who engage in social activities less than once compared to those who engage in social activities four times a week. In cases of low satisfaction with friends and community, the likelihood of cognitive impairment was 1.43 (1.29–1.58) times higher than in cases of satisfaction.

As a result of comparing the physically frail group with the robust group, the likelihood of belonging to the physically frail group increased as the number of years of education decreased, and the income level was low, particularly for those aged 74–84 and over 85 compared to those aged 65–74. Participants who experienced visual or hearing discomfort were more likely to suffer from physical frailty. As the number of concomitant chronic diseases increased, the likelihood of physical frailty also increased. Moreover, the likelihood of physical frailty increased in individuals who exhibited depressive symptoms or had IADL dependence.

As a result of comparing the cognitively frail group with the robust group, the likelihood of cognitive frailty increased in individuals aged 74–84 and 85 or older compared to those aged 65–74, and those with a low income level (Q1). The greater the number of chronic diseases, the higher the likelihood of cognitive frailty. The likelihood of cognitive frailty increased in cases of hearing discomfort, depressive symptoms, or IADL dependence. Regarding social factors, the likelihood of cognitive frailty increased 1.91 (1.33–2.75) times in individuals who engaged in social activities less than once compared to those who engaged in social activities four times a week. Older adults with low satisfaction with their friends and community were more likely to experience cognitive frailty compared to those with high satisfaction (Table 3).

## 4. Discussion

This study aimed to identify factors influencing the prevalence of cognitive frailty among Korean older adults in the community, with a focus on social factors. Our findings revealed a significant association between the frequency of social activities and subjective perceptions of friends and community, which were highly related to cognitive frailty. We discuss these points and suggest their implications.

First, it was found that the prevalence of cognitive frailty was 2.9%, while the prevalence of physical frailty was 6.1% among Korean older adults in the community. A systematic literature review of 24 studies revealed a 7% pooled prevalence of cognitive frailty among community-dwelling older adults based on descriptive studies [40]. This result was higher than the prevalence of cognitive frailty confirmed in this study. It is important to note that the prevalence of cognitive frailty may vary due to differences in diagnostic criteria and studied populations. Moreover, the variation in the physical frailty tool could account for the lower prevalence rate in this study [41]. The prevalence of frailty in Korean seniors aged 70 years or older varied from 2.5% to 12.4% depending on the scales of physical frailty [41]. As Korea rapidly transitions into an aging society, we anticipate an increase in the prevalence of cognitive frailty. Of particular interest was the finding that 47.5% of older adults with physical frailty exhibited cognitive decline, indicating cognitive frailty. This result supports the need to provide specific interventions for cognitively frail participants who have both cognitive decline and physical frailty.

Secondly, this study’s results significantly linked dissatisfaction with friends and the community to cognitive decline and cognitive frailty. These results are similar to those of previous studies showing that neighborhood cohesion has a beneficial effect on physical frailty [42] and cognitive function [43]. The elderly’s dissatisfaction with friends and the community may be a reflection of their neighborhood environment. A related study reported that neighborhood factors were independently associated with frailty in older adults [44]. Research shows that feeling more secure and having a stronger sense of social cohesion and neighborhood belonging seem to protect against frailty [42]. There is a notable gap in research regarding the conceptualization and measurement of neighborhoods and communities, as well as efforts to confirm the relationship between these entities and frailty [44]. Therefore, to identify factors of dissatisfaction with friends and the community among elderly people with cognitive decline and their caregivers, in-depth research is necessary.

Third, the results of this study indicated that older adults who engaged in social activities less than once had a higher likelihood of experiencing cognitive impairment and cognitive frailty compared to individuals who participated in social activities four or more times. This was consistent with previous research findings that the lack of social activity in the elderly is a risk factor for cognitive frailty [31], physical frailty [45], and cognitive impairment [46]. Unlike a prospective study revealing a dose–response relationship between social participation and functional decline [47], this study did not show a similar dose–response relationship between social activity and cognitive frailty. In this study, social support, defined as the number of close people and living arrangements as structural aspects of social factors, was not associated with cognitive decline, physical frailty, or cognitive frailty. Previous studies do not report consistent results regarding the relationship between social support and the health of older adults [24,48]. Furthermore, there is a lack of consistency in existing research concerning the frequency and types of social activities, as well as the multidimensional nature of social support. Given the importance of social factors in preventing cognitive frailty, it would be beneficial to conduct research that offers detailed insights into the social factors associated with cognitive frailty.

Finally, we confirmed the number of concomitant chronic diseases, depression, and dependence on instrumental daily activities as health-related factors influencing cognitive frailty. There was a correlation between the severity of physical health problems and the degree of frailty. Therefore, maintaining physical function and managing chronic diseases and mental health are important issues in frailty management for the elderly. This result reaffirmed the importance of a comprehensive approach to health issues for the elderly.

The high prevalence of cognitive frailty among participants with depressive symptoms in this study underscores the strong link between mental health and cognitive function. Research consistently identifies depression as a risk factor and a consequence of cognitive decline in older adults [49,50]. This bidirectional relationship suggests that depressive symptoms may accelerate cognitive deterioration through mechanisms such as neuroinflammation, reduced hippocampal neurogenesis, and decreased engagement in cognitively stimulating activities. Conversely, cognitive frailty can exacerbate feelings of hopelessness and diminish the quality of life. The interplay between these factors highlights the need for integrated approaches to addressing cognitive frailty. Interventions should not only aim at cognitive enhancement but also at improving mental health and emotional well-being.

It is important to help older adults with cognitive impairments remain safely at home for as long as possible [51]. According to the aging in place theory, older adults want to age in place, live with family and friends, and remain independent and autonomous; they also expect to have more opportunities to participate in social community activities, which can help them form strong senses of security and belonging to the community [52]. A study found that social isolation and loneliness, along with difficulties in daily living activities and moving around independently, are common challenges in “aging in place” among elderly individuals with cognitive impairment [51], and these findings have significant implications. The results of this study, which confirmed that participation in social activities and subjective perceptions of community influence cognitive impairment and cognitive frailty, also support the need for social intervention among older adults. Social activities have benefits for improving cognitive function [53]. The findings from Wang et al.’s [30] research revealed a connection between low social support and increased rates of cognitive frailty following a 1-year follow-up, with psychological distress partially mediating this relationship. In other words, managing mental health through social activities can help improve cognitive frailty. Social activities promote conversation, communication, and interaction between people, which can stimulate brain activity and maintain or improve cognitive abilities. This is supported by a study result reporting that deceased social interaction and instrumental social support predicted a decline in cognitive performance among older adults [48]. These findings provide insights into the mechanisms underlying the positive effects of social activities on cognitive frailty in our study. With the majority of people residing in metropolitan regions, South Korea has a substantial urban-rural split. Because of the decline of Confucianism and the transition from traditional extended families to nuclear families, older adults in urban areas frequently live apart from their offspring. This makes it difficult for them to receive direct help from family members. An absence of social interaction may be due to the fact that many elderly adults in cities live alone or in couples. Older adults living alone tend to experience faster cognitive decline than those living with others, including family members, due to a monotonous life pattern and a lack of environmental stimulation [54,55]. However, elderly residents of rural areas often have stronger ties to local norms and relationships but may have limited access to healthcare, social services, and technological advancements [56]. Therefore, health care providers need to understand regional characteristics, family resources, and social resources to improve the social activities of older adults with cognitive impairment as well as older adults with cognitive impairment. First of all, it is necessary to increase the number of centers where elderly people with cognitive decline can engage in social activities near their residential areas. It is also important to increase volunteer opportunities that help cognitively ill elderly people participate in social activities. In addition, there is a need to continue to promote the importance of social activities for the elderly with cognitive decline and cognitive frailty.

This study has several limitations. First, it only examines cross-sectional relationships between family and social factors, as well as cognitive frailty. Second, participants in the 2020 NSOK were community-dwelling older adults outside of institutional settings. As a result, the study findings may not be generalizable to institutionalized or hospitalized older adults, who may have a higher prevalence of physical frailty as well as higher rates of cognitive impairment. Despite these limitations, this study used a nationally representative sample weighted by census estimates, which increases the generalizability of these findings.

## 5. Conclusions

This study found that low economic status and health status are related not only to cognitive decline and physical frailty but also to cognitive frailty. Furthermore, vulnerability to social factors, including limited social activity or dissatisfaction with friends and community, is correlated with both cognitive decline and cognitive frailty. Therefore, we need to implement interventions that aim to enhance social engagement and the social environment for community-dwelling older adults suffering from cognitive impairment and frailty. We should actively provide opportunities for social participation and relationship building among older adults.

## Figures and Tables

**Table 1:** Participants’ characteristics and cognitive frailty (*N* = 9827).

Characteristics	Category	Total *n* (Weighted %) or M ± SD	Robust ^a^ *n* (Weighted %) or M ± SD	Cognitive Impairment Only ^b^ *n* (Weighted %) or M ± SD	Physical Frailty Only ^c^ *n* (Weighted %) or M ± SD	Cognitive Frailty ^d^ *n* (Weighted %) or M ± SD	*p* ^§^
Sex		9827 (100.0)	6945 (71.4)	2259 (21.9)	324 (3.2)	299 (2.9)	
	Male	3940 (100.0)	2723 (72.3)	961 (23.2)	91 (2.2)	95 (2.2)	<0.001
	Female	5887 (100.0)	4152 (70.7)	1298 (21.8)	233 (4.0)	204 (3.5)	
Age (years)		73.41 ± 6.53	72.76 ± 6.27	73.98 ± 6.47	78.14 ± 6.77	79.16 ± 7.16	<0.001
	65–74	5935 (100.0)	4465 (75.7)	1289 (21.4)	99 (1.6)	82 (1.3)	<0.001
	75–84	3291 (100.0)	2151 (67.1)	822 (24.0)	170 (4.8)	148 (4.0)	
	≥85	601 (100.0)	329 (56.9)	148 (22.3)	55 (8.9)	69 (11.9)	
Educational attainment (years)	(0–24)	8.44 ± 4.01	8.53 ± 4.05	8.88 ± 3.57	5.37 ± 4.45	6.29 ± 3.85	<0.001 (b > a > d > c)
Equivalent household annual income (unit: 1000 won)	Q1 (~813)	2459 (100.0)	1623 (68.3)	590 (22.5)	134 (5.3)	112 (3.8)	<0.001
	Q2 (1394)	2455 (100.0)	1628 (67.3)	639 (25.2)	85 (3.3)	103 (4.2)	
	Q3 (2435)	2452 (100.0)	1755 (70.9)	576 (23.9)	74 (3.2)	47 (2.1)	
	Q4 (2435~)	2461 (100.0)	1939 (77.9)	454 (18.6)	31 (1.5)	37 (2.0)	
Vision	Good	6594 (100.0)	4812 (73.9)	1563 (22.9)	119 (1.8)	100 (1.4)	<0.001
	Not good	3233 (100.0)	2133 (66.6)	696 (21.7)	205 (5.9)	199 (5.8)	
Hearing	Good	7563 (100.0)	5610 (75.2)	1693 (21.5)	149 (2.0)	111 (1.3)	<0.001
	Not good	2264 (100.0)	1335 (59.7)	566 (25.3)	175 (7.0)	188 (7.9)	
Depressive symptoms	No	6796 (100.0)	5191 (77.0)	1493 (21.4)	69 (1.0)	43 (0.6)	<0.001
	Yes	3031 (100.0)	1754 (59.5)	766 (24.7)	255 (7.9)	256 (7.8)	
Number of chronic diseases	(0–17)	1.83 ± 1.47	1.72 ± 1.37	1.84 ± 1.40	3.00 ± 1.97	3.22 ± 2.10	<0.001 (a, b < c, d)
IADL		10.61 ± 2.60	10.26 ± 1.60	10.68 ± 2.66	13.22 ± 5.36	15.35 ± 6.72	<0.001
	Independent	8837 (100.0)	6546 (75.0)	1996 (21.9)	176 (1.9)	119 (1.2)	<0.001
	Dependent	990 (100.0)	399 (41.8)	263 (26.7)	148 (14.1)	180 (17.5)	

Note. IADL = Instrumental Activities of Daily Living. ^§^ post hoc test by Scheffe tests (subgroups: a = robust, b = cognitive impairment only, c = physical frailty only, d = cognitive frailty).

**Table 2:** Social factors of participants and cognitive frailty (*N* = 9827).

Characteristics	Category	Total *n* (Weighted %) or M ± SD	Robust ^a^ *n* (Weighted %) or M ± SD	Cognitive Impairment Only ^b^ *n* (Weighted %) or M ± SD	Physical Frailty Only ^c^ *n* (Weighted %) or M ± SD	Cognitive Frailty ^d^ *n* (Weighted %) or M ± SD	*p* ^§^
Living arrangement	Living alone	3085 (100.0)	2073 (67.6)	728 (23.0)	145 (4.9)	139 (4.5)	<0.001
	Living with others	6742 (100.0)	4872 (72.3)	1531 (22.3)	179 (2.8)	160 (2.6)	
Social participation (days per week)	<1	2027 (100.0)	1281 (63.6)	558 (27.1)	83 (4.1)	105 (5.2)	<0.001
	1	1664 (100.0)	1177 (71.1)	365 (21.0)	59 (3.7)	63 (4.2)	
	2–3	2597 (100.0)	1902 (73.1)	582 (22.8)	72 (2.7)	41 (1.4)	
	≥4	3539 (100.0)	2585 (74.4)	754 (20.4)	110 (2.9)	90 (2.2)	
Number of closed persons	(0–87)	3.05 ± 2.56	3.13 ± 2.56	3.00 ± 2.61	2.50 ± 2.32	2.32 ± 2.27	<0.001 (a, b > c, d)
Satisfaction for friends and community	Unsatisfied	3944 (100.0)	2477 (64.0)	1063 (25.7)	191 (5.1)	213 (5.3)	<0.001
	Satisfied	5883 (100.0)	4468 (76.5)	1196 (20.2)	133 (2.0)	86 (1.3)	

^§^ post hoc test by Scheffe tests (subgroups: a = robust, b = cognitive impairment only, c = physical frailty only, d = cognitive frailty).

**Table 3:** Factors influencing cognitive impairment, physical frailty, and cognitive frailty (*N* = 9827).

Variable	Comparison (Reference Group)	Cognitive Impairment (Ref. Robust Group) Adj. OR (95% CI)	Physical Frailty (Ref. Robust Group) Adj. OR (95% CI)	Cognitive Frailty (Ref. Robust Group) Adj. OR (95% CI)
Sex Age group (years) Educational attainment	Female (male)	1.02 (0.91–1.13)	1.06 (0.78–1.44)	1.05 (0.75–1.48)
	75–84 (65–74 years)	1.31 (1.17–1.47) **	1.60 (1.17–2.19) **	1.97 (1.40–2.79) **
	≥85 (65–74 years)	1.53 (1.22–1.93) **	1.89 (1.21–2.98) **	2.84 (1.78–4.54) **
		1.08 (1.06–1.09) **	0.93 (0.89–0.96) **	1.04 (0.99–1.08)
Equivalent household annual income	Q1 (Q4)	1.50 (1.27–1.76) **	2.22 (1.40–3.51) **	2.40 (1.45–3.96) **
	Q2 (Q4)	1.71 (1.47–1.98) **	1.43 (0.92–2.24)	1.68 (1.04–2.71)
	Q3 (Q4)	1.37 (1.19–1.58) **	1.82 (1.17–2.83) **	1.30 (0.79–2.16)
Vision	Not good (good)	0.80 (0.71–1.01)	1.52 (1.13–2.04) **	1.38 (0.99–1.94)
Hearing	Not good (good)	1.32 (1.15–1.51) **	1.73 (1.29–2.33) **	2.37 (1.69–3.31) **
Depressive symptoms	Yes (no)	1.36 (1.22–1.53) **	6.02 (4.48–8.09) **	8.05 (5.62–11.54) **
Number of chronic diseases		1.01 (0.97–1.04)	1.21 (1.12–1.30) **	1.17 (1.08–1.27) **
IADL	Dependent (Independent)	1.87 (1.56–2.24) **	5.51 (4.13–7.34) **	8.30 (6.13–11.25) **
Living arrangement	Living alone (living with others)	1.02 (0.90–1.15)	0.77 (0.56–1.06)	1.01 (0.71–1.45)
Social participation (days per week)	<1 (≥4)	1.40 (1.23–1.61) **	1.31 (0.92–1.85)	1.91 (1.33–2.75) **
	1 (≥4)	1.02 (0.88–1.18)	1.36 (0.93–1.98)	1.46 (0.97–2.20)
	2–3 (≥4)	1.11 (0.98–1.26)	1.39 (0.99–1.96)	0.83 (0.54–1.28)
Number of closed persons		0.99 (0.98–1.01)	1.01 (0.97–1.05)	1.01 (0.96–1.05)
Satisfaction for friends and community	Unsatisfied (satisfied)	1.43 (1.29–1.58) **	0.99 (0.76–1.31)	1.44 (1.06–1.97) *

Note. Ref. = reference group, Adj. = adjusted, OR = odds ratio, CI: confidence interval, IADL = Instrumental Activities of Daily Living. * *p* < 0.05, ** *p* < 0.001.

## Data Availability

The original contributions presented in the study are included in the article, and further inquiries can be directed to the corresponding author.
